# Complete Genome Sequences of Three Novel Human Papillomavirus Types, 175, 178, and 180

**DOI:** 10.1128/genomeA.00443-14

**Published:** 2014-05-22

**Authors:** Hanna Johansson, Ola Forslund

**Affiliations:** Department of Laboratory Medicine, Medical Microbiology, Lund University, Malmö, Sweden

## Abstract

We report the characterization of three novel human papillomavirus (HPV) types of the genus *Gammapapillomavirus*. HPV175 and HPV180 were isolated from a condyloma. HPV178 was isolated from healthy skin adjacent to an actinic keratosis.

## GENOME ANNOUNCEMENT

We report the complete genome sequences of three novel human papillomavirus (HPV) types of the genus *Gammapapillomavirus*. HPV175 (SE87) and HPV180 (FA69) were originally detected as complete genomes in a condyloma swab sample from a 30-year-old male (1). HPV178 was isolated from a swab of healthy skin, next to an actinic keratosis of an 86-year-old male, and discovered after an attempt to amplify a closely related virus (SE46 [GenBank accession number JX198657]).

Briefly, multiple-displacement amplification (MDA)-amplified HPV DNA (1) was reamplified using the PrimeSTAR GXL DNA polymerase kit (TaKaRa Bio, Shiga, Japan). Primers were designed using the Primer Express Software v. 3.0 (Applied Biosystems). HPV175 (7,226 bp) was amplified in three parts, amplicon 1 (3,677 bp) (forward primer [fwd] 5′-ACAAATTCTCTGGGAGGACTAATGC-3′ and reverse primer [rev] 5′-GCCTCCAGTTCTTCTATAGTTCCCTT-3′), amplicon 2 (2,397 bp) (fwd 5′-CGCATGCCATGTTTGTTCTG-3′ and rev 5′-GGCCTGATTCATCTTGGATATCTT-3′), and amplicon 3 (2,601 bp) (fwd 5′-ATTTGGAAATGTTGTGATGAGTGTAAA-3′ and rev 5′-TTGCAGCCTCTCAATAGGTGTG-3′) (Eurofins MWG Operon, Germany). HPV178 (7,314 bp) was obtained as a single amplicon (fwd 5′-GTTGGTGGTGACCGAGTTTACTTT-3′ and rev 5′-TTCATTAATACAGGCCATTATAATCTACAAGT-3′) (Eurofins). HPV180 (7,356 bp) was obtained as a single amplicon (fwd 5′-TATTTGCACAAGGTGCACCAG-3′ and rev 5′-AAGGAAAGGTCAGAAAAGAGAAGCT-3′) (DNA Technology, Denmark).

Amplicons were cloned using a TOPO TA cloning kit and the pCR 2.1-TOPO vector (Invitrogen, Carlsbad, CA) and sequenced using primer walking (Eurofins).

Pairwise comparisons of the L1 open reading frames (ORF) of HPV175, HPV178, and HPV180 demonstrated closest nucleotide homology to HPV60 (68.7%, species 4), HPV65 (67.6%, species 1), and HPV121 (81.8%, species 10), respectively.

HPV175, HPV178, and HPV180 all demonstrate a typical genome organization of cutaneous HPVs, with an upper regulatory region (URR), five early genes (E1, E2, E4, E6, and E7), and two late genes (L1 and L2). In the URR of HPV175 (488 bp) we identified five consensus E2-binding sites (ACC-N_6_-GGT), one putative TATA box (TATAA), and one putative polyadenylation site (ATATAA). In the URR of HPV178 (456 bp), three E2-binding sites were present, as well as one putative TATA box and one putative polyadenylation site. In the URR of HPV180 (544 bp), we identified four consensus E2-binding sites, three putative TATA boxes, and one putative polyadenylation site.

The putative E6 proteins contained two zinc finger domains [CxxC(x)_29_CxxC] (2) separated by 36 amino acids. One zinc finger domain was also present in the E7 proteins. The LxCxE motif (binding site for the tumor suppressor retinoblastoma protein) (3) was observed in E7 of HPV178, whereas serine was substituted for cysteine in the corresponding domain (LxSxE) of HPV175 and HPV180. Among 55 E7 proteins of representative HPV types of the genus *Gammapapillomavirus*, 19 demonstrated the LxCxE motif and 27 the Lx**S**xE motif.

Only the putative E1 protein of HPV178 had the conserved ATP-binding site (GPPDTGKS) (4, 5). For HPV175 we identified GP**S**DTGKS and for HPV180 GKPNTGKS. An initiation codon of the putative start of the E4 ORF was present in HPV175 and HPV178, whereas the corresponding codon was absent in the E4 ORF of HPV180. This is in agreement with the HPV types of species 10 of the genus *Gammapapillomavirus* (6).

### Nucleotide sequence accession numbers.

The complete genomic sequences are available in GenBank under these accession numbers: HPV175 (KC108721), HPV178 (KJ130020), and HPV180 (KC108722).
